# Supplementary material for: Are carbon emissions trading and green financial instruments synergistic? -Comprehensive quantitative research based on content analysis
Source: PLoS One. 2024 Mar 7;19(3):e0298601. doi: 10.1371/journal.pone.0298601 (PMC10919601; doi:10.1371/journal.pone.0298601)
Supplement: S1 Data — (ZIP) [file pone.0298601.s001.zip › Data/地级市Z_F工作报告.zip!/╔╜╢1⁄2/┴─│╟╩╨/└┤╫╘╣1⁄2╓┌║┼í╛┬φ┐╦╩2╛▌═°í┐_2010.docx]

各位代表：

现在，我代表市人民政府向大会报告工作，请予审议，并请各位政协委员和其他列席的同志提出意见。

一、2009年工作回顾

2009年，是新世纪以来我市经济发展最为困难、极具挑战的一年，也是全市上下砥砺奋进、经受严峻考验、取得重大成绩的一年。一年来，面对国际金融危机严重冲击，我们认真执行中央、省委省政府和市委的各项决策部署，团结带领全市人民坚定信心、迎难而上、共克时艰，全力保增长、保民生、保稳定，努力推进弯道超越，圆满完成了市十五届人大二次会议确定的经济平稳较快发展、各项事业全面进步、社会保持和谐稳定的任务目标。

　　2009年，全市生产总值1375.9亿元，比上年增长13.2%；财政总收入132.8亿元，增长10.8%，其中地方财政收入55.4亿元，增长13.2%；社会消费品零售总额472.4亿元，增长19.1%；城镇居民人均可支配收入15957元，增长9.6%，农民人均纯收入5539元，增长8.4%；年末城乡居民本外币储蓄余额669.7亿元，比年初增加97.5亿元。这表明，我们取得了应对国际金融危机冲击的阶段性胜利，在建设生态型强市名城的道路上又迈出了坚实的步伐！

一年来，我们重点抓了以下六个方面的工作：

　　（一）大力开展“项目突破年”活动，项目建设实现新突破。紧紧抓住国家实行积极财政政策和适度宽松货币政策的机遇，认真落实国家刺激经济增长的一揽子计划，把开展“项目突破年”作为保增长、调结构、上水平的关键来抓，在多个方面实现了新突破。一是项目和资金争取工作实现突破。全年共争取中央和省预算内资金、省调控资金86.9亿元，为历年来争取资金最多的一年。其中，争取中央1-4批扩大内需项目330个、资金5.2亿元，均高于全省平均水平。二是项目建设规模实现突破。全市规模以上新开工项目5063个，比上年增长50%，上报和批复项目用地分别为全年指标的5.1倍和3.3倍；规模以上固定资产投资达到700亿元，增长24.9%，比全省平均水平高1个百分点左右,其中100个重点项目完成投资286.9亿元，增长29.2%。三是项目质量实现突破。全市技改投资完成159.9亿元，是去年的3.5倍；高新技术产业投资39.5亿元，增长65.8%；祥光铜业等项目达到国际一流水平。四是民生及服务业项目实现突破。全市民生工程完成投资51.5亿元，增长36.3%；20个旅游项目完成投资66亿元，增长60%；投资15亿元的盖世物流已开工建设。五是重大交通能源项目实现突破。京九铁路聊城段电气化改造全面完成，临高高速、邯济铁路扩能改造已开工建设，德商高速、济聊一级公路进入开工前准备阶段。中华电力2×60万千瓦机组已经建成，鲁皖二期成品油管道、榆济天然气管道聊城段已投入运营。

　　（二）切实加强政策引导和组织指导，保增长调结构转方式取得明显成效。坚持把保持经济平稳较快发展作为首要任务，精心组织经济运行，积极推进结构调整和发展方式转变，全市三次产业比例调整为14∶59∶27。一是积极扶持农业发展。全面落实支农惠农政策，投入扶持“三农”资金35.9亿元，增长41.7%。粮食连续七年丰收，总产468.8万吨，增长1.9%；林牧渔业产值占农业总产值比重达到28%；新增规模以上龙头企业31家、农民专业合作社858个；新增“三品”认证品种28个、基地面积41.7万亩；美国白蛾危害得到有效控制，林木覆盖率达到30%。二是扎实推进工业调整振兴。出台了促进工业企业又好又快发展的意见、加快市属企业发展的意见、九大产业调整振兴规划和节能环保产业发展指导意见，设立了1亿元的中小企业还贷周转金，积极推进银企合作，切实减轻企业负担，帮助企业渡过难关。全市新增本外币贷款177亿元，是去年的2.7倍；规模以上工业增加值、利税、利润分别增长15.9%、23.4%和26.9%；利税过千万元的企业达到400户，其中过亿元的26户。三是加快发展服务业。突出抓好商贸物流、文化旅游业发展，出台了加快旅游产业发展的实施意见，积极推进旅游、交通、物流项目建设。实现旅游总收入42亿元，增长23%；服务业增加值368.8亿元，增长12.9%。四是大力推进科技创新和节能减排。加强技术创新体系建设，推进与高校院所的合作,新增国家级企业技术中心1家、省级4家，我市被命名为有色金属材料及制品国家火炬计划特色产业基地，冠丰种业被列入国家级种质重点实验室，高新技术产业产值占规模以上工业比重提高2.2个百分点。建立健全节能降耗激励机制，鼓励引导企业建设节能减排项目，上半年全市万元GDP能耗降幅、“十一五”减排目标综合完成率由全省倒数第二位上升到中上游水平；代表山东省参加全国海河流域水污染防治工作考核，取得了第一名的优异成绩；国家环保模范城国家技术评估顺利通过。

（三）深入推进改革开放，经济发展活力进一步增强。继续深化国有企业改革，完善国有资产监管体系。稳步推进农村综合改革，农民承包土地流转27.3万亩。政府机构改革有序推进。大力发展民营经济，主营业务收入、利税分别增长21.3%和17%。继续扩大对内对外开放，在杭州、东莞、北京、西安、上海等城市和日韩、港台、俄罗斯、南美等国家和地区开展了多次招商活动，实施了“四城五楼千企工程”，与央企对接工作取得显著成效。全市招商引资到位资金202.8亿元，增长30%，引进投资千万元以上项目511个。立足于保市场、保份额，出台了稳定外需的一系列措施，进出口和利用外资降幅分别比上半年收窄8.7和60.5个百分点。积极实施“走出去”战略，信发集团印尼铝土矿开发项目和斐济并购项目取得重大进展，冠丰种业巴西棕榈系列开发项目填补了全省空白。国际友好城市工作取得新成绩。

　　（四）统筹抓好城市和新农村建设，城乡面貌发生新的变化。坚持城建靓市，进一步加大城市规划建设工作力度，全市完成城建投资51亿元，其中聊城城区25.5亿元。八大工程建设进展顺利，古城保护性整治改造有序推进，旅游接待中心主体工程已经完工，火车站改造工程顺利启动，体育公园比赛场馆、运河四期完善、生态绿化、环境综合整治、公用事业建设等工程全面完成，新增绿地面积125公顷、集中供热面积78万平方米、管道燃气居民用户9000户。城市基础设施进一步完善，建成了利民路、陈口路徒骇河大桥和东昌湖风景桥，完成了健康路、育新街等7条小街巷的改造，新增道路面积42.6万平方米。各县（市）城区建设、小城镇建设也都取得了新的进展。坚持统筹城乡发展，加快新农村建设，新建农村公路732.7公里、客运站32座，改造农村危桥223座；新建沼气池3.4万户、太阳能热水器4.7万平方米，更新省柴节煤炉灶1.6万户；新增10.2万人用上自来水；新增农村有线电视10万户，广电网络实现了农村全覆盖。大力实施农村新居建设与危房改造工程，启动整村迁建236个、千户社区建设46个，完成新居建设6.3万户、危房改造1.1万户。

　　（五）着力保障和改善民生，社会保持和谐稳定。坚持把保障和改善民生作为政府工作的着力点，切实加大对农民、城市困难群体、弱势群体的帮扶力度。积极做好就业工作，新增城镇就业9.1万人，城镇登记失业率控制在3.05%，新增农村劳动力转移就业11.8万人。加强社会保障工作，城镇各项社会保险覆盖面进一步扩大；新型农村合作医疗参合率达到97.8%，新型农村社会养老保险试点顺利启动，农村“五保”对象集中供养达到70%以上；城乡低保水平进一步提高；加大保障性住房建设力度，开工建设经济适用房10.7万平方米、廉租住房8.6万平方米，发放廉租住房补贴3022户、447.5万元。大力发展教育事业，市级财政用于教育支出2.5亿元，增长41.4%，全市新建中小学校舍9万平方米，素质教育深入实施，教学质量进一步提高；市直职业学校资源整合实现突破，组建了聊城市高级财经职业学校和高级工程职业学校，职普比例达到4∶6。加快发展卫生事业，完成了23个中心卫生院、840个村卫生室的建设或改造，城市社区卫生服务覆盖率达到100%，甲型H1N1流感、手足口病疫情得到有效防控。切实做好人口和计划生育工作，人口自然增长率控制在7‰。积极发展体育事业，成功举办了第十一届全运会火炬传递活动，圆满完成了聊城分赛区办赛工作，在全运会上取得了2金、1银、1铜的历史最好成绩；开展了“全民健身·共享全运”系列活动，“一点三线”工程建设取得重大进展。加强公共文化建设，建成乡镇综合文化站48个，中国运河文化博物馆完成布展并免费开放。深入开展社会主义精神文明建设，全社会文明程度不断提高。加强“平安聊城”建设，国庆安保、平安全运任务圆满完成，食品药品监管取得明显成效，安全事故起数和死亡人数分别下降10.46%、2.49%。积极做好援川工作，对口援建片口乡9个项目提前竣工。广播电视、新闻出版、社会科学、外侨、对台、妇女儿童、残疾人、民族、宗教、普法、史志、档案、油区、地震、气象及国防动员、人民防空、民兵预备役等工作都取得了新的进展。

　　（六）全面加强政府自身建设，工作作风明显转变。各级政府以开展深入学习实践科学发展观活动为动力，以“兴三风”为重点，加强自身建设，推进管理创新。实施了政府部门目标责任管理,健全完善目标考核和执行责任体系，强化行政问责，政府工作效能明显提高。认真落实政府依法行政情况企业评价制度和“八条禁令”，严肃查处十三种行政不作为、乱作为行为，发展环境明显改善。市行政服务中心标准化试点工作首家通过国家验收，全市共压减行政审批26项，取消或降低收费122项，减收3.3亿元。市政府所有部门实现了网上信息公开，市长公开电话承办事项办结率达到97.8%。坚持勤俭办一切事业,政府一般性开支和行政经费预算缩减了5%。健全公共事件应急预案体系，应对突发事件的能力明显增强。加强廉政建设和反腐败斗争，实行了市直纪检监察派驻机构统一管理，严肃查办了一批违法违纪案件。

　　各位代表，2009年国内外经济形势严峻复杂，农业生产先后遇到严重旱情、涝灾和低温，手足口病、甲型H1N1流感疫情接连发生，国庆安保、平安全运任务艰巨。面对巨大困难和严峻挑战，我市经济社会发展能够取得这样的成绩非常不易，这是党中央、国务院审时度势、科学决策的结果，是省委、省政府和市委科学应对、坚强领导的结果，是各级各部门齐心协力、扎实工作的结果，是全市人民团结一心、共同奋斗的结果。在此，我代表市人民政府，向全市工人、农民、知识分子、各级干部，向全市所有企业家和纳税人，向各民主党派、工商联、各人民团体和各界人士，向驻聊人民解放军、武警官兵、公安干警和中央、省驻聊单位，向关心和支持聊城发展的海内外朋友，表示衷心的感谢和崇高的敬意！

各位代表，回顾一年来的工作，我们既取得了显著的经济成果，又积累了在复杂环境中推动经济社会又好又快发展的重要经验：坚持解放思想、坚定信心，始终保持奋发有为的精神状态，牢牢把握工作的主动权，是有效应对各种危机和挑战的强大精神武器；坚持危中求机、开拓创新，创造性地开展工作，努力在关键领域实现突破，是实现弯道超越的必然选择；坚持深化改革、扩大开放，不断增强经济发展的活力，是实现科学发展的强大动力；坚持团结一心、众志成城，充分调动各方面的积极性和能动性，形成加快发展的强大合力，是实现又好又快发展的重要基石；坚持以人为本、关注民生，让广大人民群众共享发展成果，是各项事业成功的根本保证。只要我们认真发扬这些宝贵经验，求真务实，真抓实干，就一定能够战胜前进道路上的一切困难！

　　各位代表，在充分肯定成绩的同时，我们也清醒地看到，我市经济社会发展还存在不少困难和问题，政府工作也存在一些缺点和不足。主要是：受国际金融危机的影响，部分行业和企业生产经营还比较困难，经济企稳回升的基础还不够稳固；结构性矛盾仍然突出，农业产业化程度低，工业产业化链条短，服务业发展相对滞后；自主创新能力不强，节能减排任务艰巨，经济发展方式粗放；外需萎缩的局面及影响还在继续，扩大出口和利用外资面临不少困难；地方财政规模较小，结构不够合理，收支平衡难度增加；就业压力仍然较大，社会事业和社会保障等还难以满足群众的实际需要，少数群众生活仍比较困难，安全生产、社会稳定领域的问题仍时有发生；政府在规范行政行为、完善工作机制、提高工作效能等方面还有不少问题，发展环境还有待进一步改善。对此，我们一定要清醒认识、高度重视，在今后的工作中努力加以解决。

　　二、2010年面临的形势及政府工作的总体安排

　　2010年，是实施“十一五”规划的最后一年，也是应对国际金融危机冲击的关键一年。做好今年的政府工作，对保持经济平稳较快发展、为“十二五”规划启动实施奠定良好基础，具有十分重要的意义。

　　综观今年全市经济社会发展面临的形势，既有不少有利条件，也存在诸多困难和挑战。从当前的经济走势看，全球经济整体向好，国内经济持续回升，国际金融危机的影响趋于减弱，最困难的时期已经过去。但后续的深层次的影响仍然存在，全球经济复苏将是一个缓慢曲折的过程。从宏观政策和发展环境看，国家将继续实施积极的财政政策和适度宽松的货币政策，进一步充实应对国际金融危机一揽子计划，出台鼓励消费、发展战略性新兴产业的一系列措施，有利于我们进一步扩大内需、增强投资、转变方式、调整结构；省委、省政府加快建设山东半岛蓝色经济区、济南都市圈，有利于我市更好地接轨发达地区、承接产业转移；后危机时期新一轮经济结构调整已经展开，有利于我们发挥后发优势、迎头赶超、弯道超越。同时，受贸易保护主义加剧和发达经济体消费模式变化等影响，我市扩大出口和利用外资面临不少困难。从我市发展的条件看，通过开展深入学习实践科学发展观活动，广大党员干部的思想水平明显提高，科学发展的体制机制进一步完善；去年实施的“项目突破年”、调整产业结构、扩大对外开放、优化发展环境等一系列重大举措，为加快发展奠定了更加坚实的基础，经济发展后劲进一步增强。特别是经过金融危机的洗礼，全市广大干部群众团结拼搏的精神、战胜困难的信心、抢抓机遇的意识、应对复杂局面的能力显著增强。我们坚信，勤劳智慧的聊城人民不仅能经受住2009年的严峻考验，而且一定能在机遇大于挑战、有利因素大于不利因素的2010年，创造新业绩、营造新优势、实现新突破！

　　2010年政府工作的总体要求是：全面贯彻党的十七大和十七届三中、四中全会精神，以邓小平理论和“三个代表”重要思想为指导，深入贯彻落实科学发展观，认真执行中央、省委省政府和市委的决策部署，围绕生态型强市名城建设，以开展“全面提升年”活动为抓手，着力打造“一五二”产业基地，力求在抓产业、促发展上有新突破，在谋长远、抓重点上有新推进，在抓改革、促开放上有新进展，在惠民生、保稳定上有新成效，努力实现经济社会又好又快发展，全面完成“十一五”规划目标任务。主要预期目标是：生产总值增长12%，地方财政收入增长13%，全社会固定资产投资增长22%，利用外商直接投资、外贸进出口实现恢复性增长，社会消费品零售总额增长17%，城镇居民人均可支配收入和农民人均纯收入增长8%，居民消费价格指数控制在3%以内，城镇登记失业率控制在4%以内，万元生产总值能耗下降4.2%，二氧化硫和化学需氧量排放分别下降1%和1.5%,人口自然增长率控制在7‰以内。

　　各位代表，实现2010年的任务目标，必须统筹安排、科学把握：一是把保持经济平稳较快发展与加快经济发展方式转变相结合。坚持在发展中促转变，以优质的增量带动结构优化和方式转变，提高经济增长的质量和效益；在转变中谋发展，以优化经济结构、提高自主创新能力为重点，努力培植新的经济增长点。二是把城市发展与农村发展相结合。统筹工业化、城镇化、农业现代化建设，加快建立健全以工促农、以城带乡长效机制，推进城乡基本公共服务均等化，努力形成城乡一体化发展新格局。三是把发挥政府职能与发挥市场机制作用相结合。坚持用改革创新的办法解决制约发展的矛盾和问题，建立和完善政府规划引导和市场运作推进相结合的发展机制，努力为经济社会发展提供强大动力和制度保障。四是把对内开放与对外开放相结合。既要坚定不移地扩大对外开放，抓好利用外资和对外贸易；又要加强区域合作，努力承接发达地区产业转移，充分利用国内外两种资源、两个市场，加快发展步伐。五是把保障和改善民生与保持社会稳定相结合。坚持以保障和改善民生为核心，加快发展各项社会事业，及时解决影响社会稳定的突出矛盾和问题，让经济社会发展的成果更好地惠及民生。

各位代表，2010年宏观形势整体向好，但面临的形势可能会更加复杂多变。我们一定要坚持解放思想、抢抓机遇、加快发展的工作基调，千方百计加快聊城发展步伐，全面完成今年政府工作的各项任务，努力在后危机时期的竞争中赢得主动！

　　三、实施“产业提升工程”，推动二三产业实现新突破

　　在去年实施“项目突破年”的基础上，全面实施“产业提升工程”，在进一步扩张总量的基础上，提升存量、优化结构、打造特色，努力在产业规模、产业投资、产业结构、产业招商和产业集聚上实现新的突破。

　　（一）继续加大优质高效投入。把加大优质高效投入作为推进产业提升的根本，以建设“一五二”基地为目标，以重点企业为依托，着力加大投资力度、优化投资结构，不断增强产业实力。一是突出抓好项目争取工作。根据产业调整振兴规划，按照省政府确定的“八个领域”，筛选一批投资规模大、技术含量高、有利于结构升级和节能减排的重大项目，切实做好争取工作，确保争取的竞争性资金和项目达到全省平均水平以上。二是集中力量抓好重大项目建设。以先进制造业、高新技术产业、现代服务业和基础产业为重点，继续抓好100个重点项目建设，完善组织领导、部门帮包和考核奖惩制度，加大政策扶持力度，确保项目建设扎实推进。抓好中央新增投资项目建设，确保按时保质保量完成任务。三是积极谋划新的优势产业项目。按照“三个一批”的要求，立足产业和资源优势，继续抓好重大优势产业项目的筛选、论证和申报工作，为聊城的长远发展提供支撑。

　　（二）努力推进工业调整振兴。把工业经济作为产业提升的重中之重，按照“转方式、调结构、塑特色、扩总量”的工作思路，大力实施“双千翻番工程”，用三年左右的时间，实现有色金属及深加工产业产值过千亿元，规模以上企业增加1000家，规模以上企业销售收入、利税、上缴税金翻番。2010年，有色金属及深加工产业产值达到700亿元。一是做大做强优势产业。集中力量发展有色金属及深加工产业，突出抓好铜、铝两个千亿产业聚集区和精深加工产业园建设，向精深加工和高端产品延伸产业链条，努力把我市建成全国重要的有色金属生产及深加工基地。大力推进运输设备及零部件、基础化工及精细化工、轻纺造纸及中医药、能源电力及节能设备等基地建设，扩大产业规模，完善产业链条，促进集群式发展。二是积极培育战略性新兴产业。制定和实施培育战略性新兴产业总体规划，强化政策支持和财政投入，突出抓好新能源汽车、鲁西化工产业园和节能环保产业的发展，力争高新技术产业产值占规模以上工业比重提升2个百分点。三是加快传统产业升级改造。采取政府采购、风险补助等措施，鼓励企业采用高新技术对产品设计、生产制造、市场营销等环节进行技术改造，提高工艺装备水平和附加值。推进信息化和工业化融合，支持企业广泛运用信息技术，提升自动化、智能化和管理现代化水平。引导企业创新管理模式，降低生产成本，提高运行质量。四是继续抓好大企业发展。以主营业务收入前50户企业为重点，开展创新进档升级活动，大力推进技术创新、管理创新和制度创新，努力把企业做强、做稳、做到可持续发展。高度重视市属企业发展，找准发展方向和路子，积极上项目、上规模、上水平。五是支持和引导中小企业发展。搞活民间投资，鼓励全民创业，特别是围绕优势产业前延后伸，发展特色产业园区和特色产业乡（镇）村（居），培植新的产业集群。继续实施“双百工程”，抓好16处小企业创业辅导基地建设。加强信用担保体系建设，健全完善中小企业还贷周转金，努力解决中小企业融资难问题。

　　（三）加快发展服务业。坚持三产兴市，把提高服务业在国民经济中的比重作为产业提升的重要内容，以商贸物流业、旅游度假业为重点，加快服务业发展步伐。一是突出发展商贸物流业。加快聊城物流中心建设，全面推进铁路物流区、国际物流区、农产品物流区、仓储配送区、综合服务区等五大规划分区建设，积极引进国内外知名物流企业，打造冀鲁豫交界地区综合物流服务枢纽。积极发展第三方物流，推动主辅分离企业从服务主业向为社会服务方向转变。加快完善市场体系，推进临清烟店轴承、大东钢管、香江等市场的改造升级。建立健全城市超市连锁经营体系，抓好聊城银座商城、新东方国际等大型商业网点建设。继续实施“万村千乡”和下乡产品流通网络工程，构建农村现代商品流通体系。二是大力发展旅游度假业。以打造“江北水城·运河古都”为目标，坚持政府主导、社会参与、项目带动、市场突破，尽快把旅游业培育成战略性支柱产业。集中力量抓好水上古城保护性整治改造、徒骇河世界运河（建筑）博览园、马颊河生态旅游度假区、阿胶养生文化苑、水浒文化旅游、蚩尤陵园等项目建设，打响“中华水上古城”品牌。发挥温泉资源优势，整合农业、林业、文化等资源，规划建设以温泉为特色的城乡旅游休憩带。加强旅游资源整合，积极培育旅游骨干企业。继续举办“五一”、“十一”等特色旅游节庆活动。加强与济南都市圈、中原地区、京津地区及长三角等重要旅游板块的合作,扩大客源市场。三是积极发展其它服务业。按照面向市场、以销定建原则，合理调控房地产开发规模，重点发展面向中低收入阶层的普通商品房、经济适用房和廉租住房，推动房地产业健康发展。把金融业作为重要的产业来抓，积极引进外地股份制银行，加快培育村镇银行、小额贷款公司、农村资金互助社，规范发展多种形式的保险公司，支持企业直接上市融资，扩大企业债券融资规模。实施重大文化产业项目带动战略，加快文化产业基地和区域性特色文化产业群建设，培育龙头文化企业和特色文化品牌，发展游艺、动漫、影视、网络等新兴文化产业。加快发展信息服务、社区服务、中介服务等服务业。四是认真落实消费政策。简化手续，完善网络，进一步做好家电、汽车、摩托车下乡工作，继续实施家电和汽车以旧换新政策，为方便和扩大居民消费创造更加宽松的环境条件。

　　（四）切实抓好科技创新和节能减排。坚持把科技创新和节能减排作为产业提升的主攻方向。一是大力推进科技创新。着眼于提高聊城未来竞争力，努力打造一流的高新技术研发平台，力争全年新增省级以上工程技术研究中心15家、技术中心4家；鼓励、引导有实力的企业牵头，成立产业技术创新战略联盟，建立公共技术平台。抓好西安交大聊城科技园建设，推动企业同高校共建工程专业和实训基地，进一步拓展同高校院所的科技合作与交流。加强创业服务中心建设，建立健全中介服务、公共技术服务、快速制造技术服务、金融服务四大平台，进一步提高服务效能。加快知识产权交易平台建设和专利技术推广应用。加强科技人才队伍建设，培养好现有人才，引进更多高端和企业紧缺人才。二是切实抓好节能减排。继续抓好50户重点用能企业和130户重点排污企业节能减排工作，加快推进循环经济“2320”工程，大力推行清洁生产。积极发展生态经济，实现绿色发展。全面推进节约用水，抓好南水北调沿线、马颊河、徒骇河、卫运河流域工业点源和城市污染源治理，新增城市污水年处理能力1000万吨，扩建垃圾处理项目3个。抓好燃煤电厂脱硫工程建设，搞好城市扬尘、工业废气和机动车尾气治理，进一步改善空气质量。全面完成国家环保模范城创建工作，并以此为契机，努力使我市节能减排工作登上新的台阶。

　　四、夯实“三农”发展基础，促进农村发展农民增收

　　坚持三农稳市，把“三农”作为全市经济社会发展的重中之重，以增加农民收入为中心，采取有效措施，加快农业和农村经济发展步伐。

　　（一）深入推进农业结构调整。以构建现代农业产业体系、打造生态农业及农产品深加工基地为目标，制定和实施农业产业调整振兴规划，重点实施“四百工程”，即：在三到五年内，实现蔬菜和食用菌产值100亿元、畜牧和水产产值100亿元、林果生产及加工产值100亿元、粮食生产能力突破100亿斤。严格执行国家粮食直补、农资综合补贴、良种补贴、农机购置补贴等强农惠农政策。开展粮食高产创建工程，在不增加种植面积的前提下，尽快实现粮食生产能力过100亿斤。引导农民积极发展蔬菜、食用菌、畜牧、林果等高效产业，着力建设八大设施蔬菜示范基地、六大特色果品生产基地、五大优势畜产品生产基地，提高农业的专业化、规模化和集约化水平。大力推进农业产业化，加大对龙头企业扶持力度，力争规模以上龙头企业达到350家，粮食加工转化率每年提高3个百分点；发展农民专业合作社1500家以上。积极开展植树造林，抓好美国白蛾防控，全市林木覆盖率提高1个百分点。抓好市级农产品综合批发市场建设，推进“农超对接”，努力开拓农产品市场。进一步健全农业生产标准体系和农产品检测体系，力争新增“三品”认证品种18个、基地面积10万亩。深入实施农业科技示范工程和科技特派员工程，加强农民实用技术培训，为农业结构调整提供科技支撑。继续实施沃土工程和清洁工程，开展测土配方施肥和秸秆还田、秸秆综合利用，推进农业可持续发展。

　　（二）加强农村基础设施建设。重点实施五大工程：一是农田水利建设工程。抓好南水北调工程聊城段和位山、郭口、彭楼三处引黄灌区续建配套工程建设，搞好平原水库建设，发展节水灌溉面积4万亩，新打机井2000眼、修旧3000眼。二是农村交通建设工程。新建农村公路350公里，改造农村危窄桥30座，力争实现所有行政村通柏油路。三是农村通自来水工程。新增10万人用上卫生安全自来水。四是农村通有线电视工程。新增有线电视4万户。五是农村可再生能源开发利用工程。推广农村户用沼气池2.5万个、太阳能热水器2.5万户，年减排二氧化碳4.65万吨。

　　五、进一步加强城乡建设，加快新型城镇化进程

　　加快新型城镇化进程，是扩大内需、保持经济平稳较快发展的战略举措。要进一步强化城镇集群发展、城乡统筹发展、城乡可持续发展“三个”理念，启动和实施百亿城建、百亿新居、百亿产业“三个百亿元工程”，确保全市城镇化率每年提高2个百分点、力争3个百分点，2020年力争达到全省平均水平。

　　（一）突出抓好城市建设。坚持高起点定位、高水平规划、高标准建设、高效能管理，着力拉大城市框架、完善城市功能、提升城市品位，为城镇化提供坚实基础。集中力量抓好中心城市建设，按照“一城五区”的总体布局，以徒骇河为中轴，大力实施“两城一河”发展战略，努力实现“一年一变化，五年大变样”，使中心城区建成区面积逐步达到100平方公里以上、人口100万以上。今年，重点实施“十大工程”：一是古城保护性整治改造。完成城墙临水四边和四关大街建设，2011年基本形成古城框架。二是徒骇河世界运河（建筑）博览园建设。搞好规划设计，启动重点项目，拉动沿线开发。三是工业新城开发工程。抓好开发区会展中心、中央商务区、干线道路等项目建设，推动工业新城快速崛起。四是城市基础设施工程。抓好城区道路、桥梁、节能灯改造等项建设。五是文体旅游工程。抓好市民文化艺术中心、体育公园、旅游接待中心、益华广场及温泉度假村等项目开发。六是民居改善工程。启动20个城中村改造。七是科技教育工程。加快高新技术园区规划建设，实施西安交大聊城科技园建设，推进高级财经职业学校、高级工程职业学校建设。八是创建国家园林城市工程。实施主次干道增绿升级、节点绿化和运河景观升级，建设一批街头绿地（游园）、城市公园、城市雕塑。九是水系完善工程。实施运河五期、运河六期、南关岛和三山岛开发，运河与徒骇河连通及湖南湖、东南湖整治工程。十是商贸物流工程。包括聊城物流中心、市级农产品综合批发市场建设等。加强县（市）城区建设，依据发展定位，按照功能分区，确定突破重点，打造一批商贸新区、文化新区、居民新区、工业新区，全面提升建设水平。抓好城市建设资金筹集，在引进有实力的大财团、大开发商来聊投资的同时，探索组建城市建设投资开发公司，打造政府筹集资金、建设城市的新型平台。进一步理顺城市管理行政执法体制，创新执法模式，优化城市环境，提高文明程度。

　　（二）积极推进城乡一体化。坚持统筹城乡发展，进一步完善规划，加大投入，以“路、水、电、气、医、学”为重点，推动基础设施向农村延伸、社会事业向农村覆盖、公共服务向农村辐射。抓好中心镇、中心村建设，积极稳妥地推进村庄合并，启动100个千户社区建设。充分发挥工业园区的辐射带动作用，探索“村企合一”新路子，争取两年内完成76个工业园区村的改造建设。用足用好增减挂钩政策，切实维护农民利益，抓好农村新居建设和危房改造，建设农村新居10万户，改造农村危房1万户。我们要逐步让越来越多的农民住上与城里人基本一样的新房子！

（三）加快构建综合交通体系。立足于山东东引西拓“桥头堡”的战略定位，着力完善交通枢纽功能，重点实施“双四一”工程，即铁路建设实施“四路一站”工程、公路建设实施“四路一枢纽”工程。抓好聊城火车站改造、邯济铁路扩能改造，推进济南至聊城城际轨道交通、聊泰铁路项目启动。加快临高高速公路建设，力争德商高速、济聊一级公路开工建设，完成泰安至聊城高速前期工作。以市城区为中心，抓好干线公路综合整治和改建，推进县城和乡镇路网扩容提速，打造便捷、高效、通畅的“半小时经济圈”。

　　六、坚定不移地深化改革扩大开放，努力为经济社会发展提供强大动力

　　改革开放是经济社会发展的根本动力。要通过深化改革激发内在活力，完善有利于科学发展的体制机制；通过扩大开放汇聚更多的经济要素，在融合与竞争中营造出竞争发展的新优势。

　　（一）积极推进重点领域和关键环节的改革。一是推进国有企业改革。继续推进国有企业股份制改革，规范和完善企业法人治理结构，选择有条件的企业开展引进外部董事试点。建立困难企业重组脱困机制，做好企业破产处置和职工安置工作。依法加强国有资产监管，完善国有资本经营预算制度。二是推进农村改革。建立健全土地承包经营权流转市场，按照依法自愿有偿原则，鼓励扩大土地流转，发展适度规模经营。深化集体林权制度改革，明晰产权，搞活经营，调动农民生产积极性。积极推行农村小型基础设施产权改革，建立完善农村公路、自来水等基础设施养护管理体制。三是推进政府机构改革。在抓好市级政府部门“三定”工作的同时，加快推进县（市、区）政府机构改革，着力转变政府职能，理顺职责关系，提高行政效能，为经济社会发展提供体制机制保障。四是推进其它各项改革。深化财税体制改革，完善财政奖励补助政策和县乡财政管理体制，全面推进国库集中支付制度，开展公务卡结算改革试点。推进事业单位改革，全面实施人员聘用制度和公开招聘制度，加快经营性文化单位转企改制。有计划、有步骤地放宽中小城市户籍限制，促进城镇化发展。扎实推进医药卫生体制改革，加快建立覆盖城乡居民的基本医疗卫生制度。

　　（二）全面扩大对内对外开放。一是大力开展招商引资。坚持招商引资“一把手”负责制，以政府为主导、企业为主体、产业为依托、项目为抓手，进一步加大工作力度，确保全年实现招商引资到位资金230亿元。继续推进同长三角、珠三角、京津地区的合作，主动对接山东半岛蓝色经济区，加快融入济南都市圈，千方百计争取发达地区产业转移。深化同日韩港台的合作，加大对欧美招商引资工作力度，着力引进先进制造业、现代服务业和高新技术产业项目，提升利用外资水平。精心组织好“2010海内外知名企业家齐鲁行”活动，深入实施“四城五楼千企工程”，积极推动与世界500强、大型央企和国内外知名企业的合作。突出抓好产业招商，深入开展网络招商、节会招商、委托招商，推动大企业集团销售网络招商，及时帮助客商解决困难，促进以商招商。突出工业园区对外招商的平台作用，举全市之力加快经济开发区发展，启动九州高科技园建设，打造全市经济又好又快发展的最优平台和最快增长点。二是努力扩大对外贸易。实施市场多元化战略，积极组织企业参加境内外展洽活动，鼓励企业建立境外自主销售网络，着力巩固传统市场，积极开拓新兴市场。支持优势企业、出口大户、重点产品扩大出口。调整优化出口商品结构，扩大自主知识产权、自主品牌、自主营销和高技术含量、高附加值、高效益产品出口。做好对接青岛保税港区工作，建立出口企业服务体系，完善反倾销应诉支持机制和出口产品预警机制，帮助企业化解出口风险。支持企业进口先进技术设备、关键零部件和重要原材料，增加战略性资源储备。三是积极实施“走出去”战略。用足用好扶持政策，推动骨干优势企业建立境外生产基地，探索以“工程换资源”等新型对外承包形式，带动资本、产品、劳务输出。实施资源供应多元化战略，抓好境外铝土矿、天然橡胶、木材、铜精矿和棕榈油五个基地建设，使基地资源进口量逐步达到实际需要量的30%以上。

　　七、切实保障和改善民生，促进社会和谐稳定

　　坚持把保障和改善民生作为政府工作的着力点，作为扩大内需、推动发展方式转变的重大举措，坚定不移地加以推进。

　　（一）努力扩大就业。实施积极的就业政策，突出解决好重点人群就业问题。一是做好城市弱势群体就业工作。开展“千个公益性岗位开发助特困就业”活动，对“4050”人员、零就业家庭成员、残障人员等进行“一对一”帮扶。认真落实小额担保贷款政策，以创业带动就业。二是做好高校毕业生就业工作。认真落实“七项措施”，鼓励自谋职业、自主创业，确保当年高校毕业生就业率达到70%以上。三是做好农村劳动力就业工作。加快建立服务、培训、维权三位一体的农村劳动力输出工作机制，大力发展劳务派遣和境外劳务，促进返乡农民工二次就业或自主创业，新增农村劳动力转移6.5万人，在外务工人员保持在110万人以上。

　　（二）健全城乡社会保障体系。一是加快建设农村社会保障制度。全面落实农村“五保”供养政策。深入扎实地开展新型农村社会养老保险试点，加大资金投入，完善配套措施，确保试点成功。巩固和完善新农合制度，参合率稳定在95%以上，人均筹资水平提高到120元。二是完善城镇社会保障制度。抓好企业职工基本养老保险省级统筹，提高企业退休人员基本养老金水平，逐步解决城镇未参保居民养老保障问题。加快推进城镇职工、城镇居民医疗保险，尽快实现市级统筹。加强社会保障扩面征缴，着力抓好非公有制经济从业人员、农民工和灵活就业人员参保工作。加快保障性住房建设，开工建设经济适用住房10万平方米，新增廉租住房300套、廉租住房租赁补贴500户，改造棚户区3200套。三是健全城乡社会救助体系。继续实施城乡低保标准联动机制，进一步提高低保水平，农村低保标准提高到1200元以上。实施贫困残疾儿童康复救助计划，加大对残疾人生活救助力度，完成农村贫困残疾人危房改造200户、1万平方米。加强县级社会福利服务中心建设，新建老年服务机构5个。完善城乡医疗救助体系，放宽病种限制，取消救助起付线。强化救灾减灾工作，妥善安排好灾民生活。

　　（三）加快发展各项社会事业。一是优先发展教育事业。加快实施城区中小学改扩建工程，解决小学班额过大问题；实施农村中小学仪器更新工程和“两热一暖一改”工程，努力改善办学条件。加强学校规范化建设，推进素质教育，提高教育质量。大力发展职业教育，做好市直职业教育资源整合后续工作，优化高中和中职在校生比例。支持发展特殊教育。加强地校合作，支持聊城大学、聊城职业技术学院、东昌学院和技师学院的发展。二是繁荣发展文化事业。加强社会主义核心价值体系建设，扎实推进理想信念和思想道德教育，深入开展文明城市、文明村镇、文明行业等群众性精神文明创建活动。建设市群众文化艺术中心，加快县（市、区）图书馆、文化馆建设进度，全面完成乡镇综合文化站建设任务。理顺广电网络管理体制，加快城区数字电视整体转换。积极实施“文化精品工程”，广泛开展形式多样、丰富多彩的群众文化活动。加强文化遗产的保护管理利用，做好运河文化遗产资料的整理和保护规划的编制实施。三是加快发展卫生事业。加强城市社区卫生服务机构建设，全面完成村卫生室规划建设任务。制定完善常见病诊疗规范，注重中医药适用技术推广应用，让群众享受更为便捷安全的医疗服务。高度重视甲型H1N1流感、手足口病等传染性疾病的预防控制，提高突发公共卫生事件应急处理能力。四是积极发展其它社会事业。坚持人口与计划生育基本国策，优化出生人口结构，稳定低生育水平。积极发展体育事业，加快推进体育公园建设，支持发展竞技体育，实施全民健身计划，着力打造“运动之城、健康之城、阳光之城”。加强国防建设，抓好双拥和优抚安置政策落实，争创全国全省双拥模范城“三连冠”。继续做好援川工作。关心支持老龄、妇女儿童、残疾人事业，进一步做好新闻出版、社会科学、民族、宗教、对台、侨务、油区、人防、档案、史志、气象、测绘、防震减灾等项工作，促进社会事业全面发展。

　　（四）全力维护社会稳定。深入开展普法教育，引导群众依法表达利益诉求。坚持领导干部接访制度，认真做好群众来信来访工作，及时合理解决群众反映的问题。完善以预案和体制、机制、法制等“一案三制”为内容的应急管理体制，健全社会稳定预警机制，积极预防和妥善处理各类突发公共事件。深入开展“平安聊城”创建活动，加大对未成年人犯罪的预防力度，加强社会治安综合治理，依法严厉打击各类违法犯罪活动。深入开展“安全生产基层基础年”活动，抓好重点行业、重点领域、重点时期专项整治，努力消除安全隐患。全面整顿和规范食品药品市场秩序，建立健全防控体系。我们要尽最大的努力，确保全市人民身体健康和生命安全，不受假冒伪劣产品的侵害！

　　八、大力推进管理创新，提高政府服务水平

　　各位代表，今年我们面临着推动发展与自身改革的双重任务。我们要坚持以改革促发展，大力推进政府管理创新，着力建设服务政府、责任政府、法治政府和廉洁政府，为经济社会发展营造良好的政务环境。

　　（一）转变政府职能，建设服务政府。把转变政府职能作为机构改革的核心，加快推进政企分开、政资分开、政事分开、政府与市场中介组织分开，更大程度地发挥市场在资源配置中的基础性作用。深化行政审批制度改革，进一步清理和规范行政审批事项，确保政府机构改革中要求取消、下放、转移的事项落实到位。加强市、县两级行政服务中心建设，推进行政审批集中办理。强化服务意识，经常深入基层、深入实际，加强调查研究，积极为群众、为企业排忧解难。抓好市长公开电话和行风热线建设，加强直接面向基层和群众的服务，使广大群众真正从改革中得到便利和实惠。

　　（二）改进管理体制，建设责任政府。坚持用改革创新的办法解决制约发展的问题，积极探索有利于科学发展的新思路、新举措。完善政务公开制度，改进政府公报、政府网站、新闻发言人等公开渠道，着力推进政府信息公开、决策公开、办事公开和内部公开，努力实现行政权力公开透明。坚持政府部门目标责任管理，探索实行政府绩效考评制度，健全完善评估体系，改进考评方式，注重考评结果的使用，推动政府在各个环节都能合理高效地配置和使用资源，降低行政成本，提高行政效率。着力推进行政问责的制度化、规范化，加大对行政不作为和乱作为的问责力度。深入开展“优化发展环境回头看”活动，完善“三本”备案备查和企业评价制度，严格落实“八条禁令”，严肃执行各项纪律，加强监督检查，努力营造春天般的发展环境。

　　（三）严格依法行政，建设法治政府。深化法治理念，自觉按照法定的权限和程序行使权力、履行职责，主动接受人大及其常委会的工作监督、法律监督和政协的民主监督，高度重视新闻舆论监督和社会公众监督，支持监察、审计部门依法履行监督职责。严格依法办事，切实保护公民、法人的合法财产和正当权益。加强行政执法队伍建设，提高行政执法人员的整体素质和执法水平。加大执法监督力度，全面推行行政执法责任制，加强行政复议和执法监督，完善执法评议考核、行政赔偿和过错追究制度，形成全方位、全过程、有重点的监督体系。

　　（四）坚持从严治政，建设廉洁政府。严格遵守《党员领导干部廉洁从政若干准则》，认真落实领导干部党风廉政责任制，规范各级干部的从政行为。加大从源头上预防腐败的力度，抓住规范权力运行、正确行使权力这一关键，加快建立电子政务服务、行政审批、公共资源交易、公共资金监管、舆论监督、廉政教育六大电子监察平台，加强对政府关键岗位、重点领域、重点环节的监督制约，强化对财政资金和重大投资项目的监察审计。进一步转变作风，大兴调查研究之风、干事创业之风、狠抓落实之风。自觉树立过紧日子的思想，坚决杜绝形式主义和各种铺张浪费现象。深入开展专项治理，坚决纠正损害群众利益的不正之风，依法查处违法违纪案件和损害发展环境的行为，全面提升政府公信力。

　　各位代表，国际金融危机的影响尚未结束，后危机时期新一轮发展与竞争已经展开。面对新形势、新机遇、新挑战，我们一定要以只争朝夕的精神、大胆探索的勇气、高度负责的态度，踏踏实实地干好工作，努力向全市人民交一份合格的答卷！让我们更加紧密地团结在以胡锦涛同志为总书记的党中央周围，坚持以邓小平理论和“三个代表”重要思想为指导，深入贯彻落实科学发展观，在市委的坚强领导下，解放思想，锐意进取，扎实苦干，推动聊城在加快建设生态型强市名城的征程上，迈出新的更大的步伐！
